# Keeping medical information safe and confidential: a qualitative study on perceptions of Israeli physicians

**DOI:** 10.1186/s13584-024-00641-9

**Published:** 2024-09-27

**Authors:** Keren Semyonov-Tal

**Affiliations:** 1 Department of Labor Studies, Tel Aviv University, Tel-Aviv, Israel; 2 Department of Sociology, Institute of Sociology and Social Psychology University of Cologne, Cologne, Germany

**Keywords:** Medical confidentiality, Physician’s perspectives, Qualitative methods, Israeli Healthcare system

## Abstract

**Background:**

Patients expect their information to remain confidential, and physicians have a legal and ethical obligation to keep it this way. Confidentiality is not just a legal requirement but a crucial element in establishing trust between patients and healthcare providers. Patients must feel confident that their personal and medical information is kept confidential and shared only with those who need to know. Previous studies have primarily concentrated on patients’ perceptions of medical confidentiality, data privacy, and data protection issues. However, research on the practical practices and perceptions of medical confidentiality among hospital physicians is scant, underscoring the need for a deeper understanding of this critical issue.

**Methods:**

Through qualitative methods and as part of a large-scale study on aspects of patient dignity and responsiveness in Israel, physicians shared their views and practices on managing medical information.

**Results:**

The study revealed the practical challenges physicians face in upholding various aspects of data protection within hospital settings. These challenges, strategies, and deviations from data protection principles that physicians discussed are of significant practical relevance. The importance of patient consent and the practical measures for safeguarding patient information were also highlighted. While physicians acknowledged the importance of protecting patient information, they also grappled with the realities of doing so in a complex healthcare environment. In future healthcare policies, it is critical to ensure robust measures are in place to safeguard and uphold medical confidentiality. These can include specific measures to increase compliance, such as regularly monitoring compliance with confidentiality policies, producing safe and anonymous channels to voice concerns, and enforcing consequences for any breaches to ensure accountability.

**Conclusions:**

While protecting medical information has emerged as an important goal, it is equally crucial to strike a balance between the need to share information to advance and provide quality medical care. Physicians and policymakers must navigate this delicate balance. Additionally, organizations should strengthen compliance to enhance their monitoring and enforcement of confidentiality policies. Ineffective implementation of medical confidentiality leads to theoretical guidelines that do not translate effectively into practice.

## Introduction

Medical confidentiality is a cardinal aspect of healthcare responsiveness and patient dignity. Patients must feel assured that their personal and medical information is kept confidential and only shared with those who need to know [1, 2]. The doctor-patient relationship is built on trust, and medical confidentiality ensures that doctors can maintain such trust by protecting the privacy of their patients [3]. Sharing medical information without prior consent breaches confidentiality [3, 4]. However, it is difficult to maintain medical confidentiality in modern hospitals due to multiple medical teams’ involvement and electronic advancements [5–7]. Different primary healthcare team members may require access to patient medical information [8]. In some cases, and under specific circumstances, family members may also require access to medical information. For example, doctors may need to share information with other healthcare professionals involved in the patient’s care or with family members with a legitimate interest in the patient’s health [9]. On the other hand, there are instances where doctors must protect the medical information and cannot disclose it to anyone else. For example, if a patient explicitly requests that their medical information be kept private, doctors should respect that request unless there are legal or ethical reasons to disclose the information [4].

## Background

### Responsiveness of care and patient dignity

Responsive healthcare refers to how patients are treated and their experiences when interacting with the healthcare system [10–12]. In other words, healthcare responsiveness pertains to how well services meet patients’ needs and preferences [13]. From this perspective, a responsive healthcare system improves patients’ health outcomes and well-being [14]. According to the World Health Organization, responsiveness in healthcare involves treating patients with dignity, maintaining strict confidentiality of their medical information, ensuring effective communication between patients and healthcare providers, respecting autonomy, providing timely medical attention, granting access to social support, offering basic yet high-quality facilities, and allowing patients to choose their healthcare provider [13, 15, 16].

Confidentiality is a major and crucial component of patient dignity. For example, Matiti (2002) [17] defines dignity in medical settings as comprised of several important components, including confidentiality, access to information, and respect.

### Patient perceptions of confidentiality

When discussing medical confidentiality, patients express the need for (a) policies to require providers to explain procedures for sharing information, (b) obtain patients’ specific consent for access to their medical records, and (c) impose penalties on those who breach confidentiality [4]. According to a comprehensive literature review conducted by Moran and colleagues in 2003 [1], patients expressed three main concerns regarding confidentiality. First, they were concerned about physicians sharing medical information with other physicians. Second, they were concerned about physicians sharing medical information with family members and others. Third, they expressed the need for understanding and specifying the circumstances under which physicians can breach confidentiality.

Indeed, patients acknowledged the need for physicians to share information with other medical personnel involved in their care [18]. Likewise, patients mostly agreed to share their medical information with family members [9]. Nevertheless, sharing information with others is perceived as more complex [19]. Hence, patients’ perceptions of confidentiality breaches varied depending on the type of information disclosed and the recipient of the information [1].

### Physicians’ perceptions of confidentiality

Preserving patient confidentiality is a fundamental aspect of healthcare practice, emphasized in the Hippocratic Oath [2]. According to a study conducted by Barnie (2015) [20], health workers have a high regard for protecting confidentiality and medico-legal issues. Physicians are legally and ethically obligated to protect patients’ data [21]. Protecting patients’ medical information enables patients to seek medical care without fear of harm or embarrassment, ultimately enhancing the quality of treatment [13].

Despite the general and wide agreement about the importance of data protection, patient data privacy breaches still frequently occur in hospital settings. A study conducted in a university ED revealed that all healthcare team members violated confidentiality and privacy policies [22] and that the violations were associated with the hospital’s infrastructure. Violations were mostly observed in public areas such as meeting rooms, examination rooms, nursing stations, rooms occupied by more than one patient and companions, corridors, and elevators [23, 24]. The breaches included disclosing patient data to unauthorized third parties, discussing patient information in public areas, improperly disposing of patient records, leaving electronic or paper health records unattended, and providing care with open doors [23].

However, not all physicians agree with the rule of keeping medical information confidential in all situations. For example, local Israeli law allows some breaches of confidentiality in order to protect public safety or patient well-being (for the Israeli rules and regulations on medical confidentiality, see [25, 26]). Studies also indicate that physicians often have divergent views on whether and when it is appropriate to breach patient confidentiality. While some physicians consider confidentiality an absolute, unbreakable rule, others attempt to balance confidentiality with the need to prevent harm to others or themselves [27, 28]. Hence, physicians’ perspectives and practices related to confidentiality can differ. Likewise, strict adherence to confidentiality regulations may conflict at times with other ethical responsibilities or concerns for public safety. However, it seems that certain thresholds for breaking confidentiality, which typically center around preventing harm to the patient or to others, can be identified [7) Moreso, there are scenarios where more comprehensive information is provided could lead to better patient care and a more responsive system, even if not fully secured [10–12]. For example, quick access to a patient’s full medical history, including potentially sensitive information, could be lifesaving in emergency situations (for a literature summary, see Appendix C).

## Research question

Although medical confidentiality is a well-researched topic, previous studies focus mostly on patient perceptions, legal policies, and the need for policy reforms and data privacy protection. Only very few studies have examined physicians’ perceptions of medical confidentiality in hospital settings and obstacles to its preservation (for notable exceptions, see [27, 28]). Therefore, the present study seeks to contribute to the literature on medical ethics by outlining physicians’ perceptions of medical confidentiality and the challenges physicians face in upholding data protection and preserving confidentiality within hospital settings. Based on the literature reviewed and presented in the previous sections, I arrived at the following three working hypotheses:

### Hypothesis 1

Whereas some physicians are likely to view medical confidentiality as an absolute rule, others may consider breaching the rule under certain circumstances [27, 28].

### Hypothesis 2

Modern hospitals’ infrastructure and practices (e.g., open areas, electronic records, multiple team involvement) are likely to pose significant challenges to maintaining patient confidentiality, leading to frequent breaches by healthcare professionals [23, 24].

### Hypothesis 3

Hospitals’ infrastructures and structural constraints are likely to limit physicians’ ability to safeguard confidentiality [22–24].

## Method

Twenty in-depth semi-structured interviews with physicians employed in Israeli public hospitals were conducted to address the literature gap (for demographic information, see Appendix Table A). Most interviews were conducted over Zoom in 2020 due to COVID-19 restrictions and were audio-recorded and transcribed to ensure anonymity. Participants were recruited using personal connections and snowball sampling (asking interviewees to connect with other potential participants who met the criteria) [29]. This resulted in a diverse group of physicians with varying seniority and experience. The author conducted all the interviews. The participants were provided with an information leaflet, which explained the study and addressed issues of confidentiality and anonymity. Informed consent was obtained from all participants. The University’s ethics committee approved the study. A thematic analysis of the interviews was conducted. Data analysis followed Braun and Clarke’s [30] thematic method, which involves a deep exploration of qualitative data to develop codes and themes. Throughout the research process, the author took manual analytical notes. During the coding phase, surface meaning and interpretive codes were used to capture participants’ perceptions of medical confidentiality. The coded data was then organized into potential sub-themes and themes. These codes and themes were discussed with colleagues as experts and, as a result, refined into four themes. In the final phase, the author reviewed the interviews to ensure that the codes and themes formed a consistent and logical pattern. Finally, the author documented the final findings using themes, relevant analytical notes, and data excerpts to ensure the transferability and credibility of the findings^1^.

## Results

**Fig. 1:**
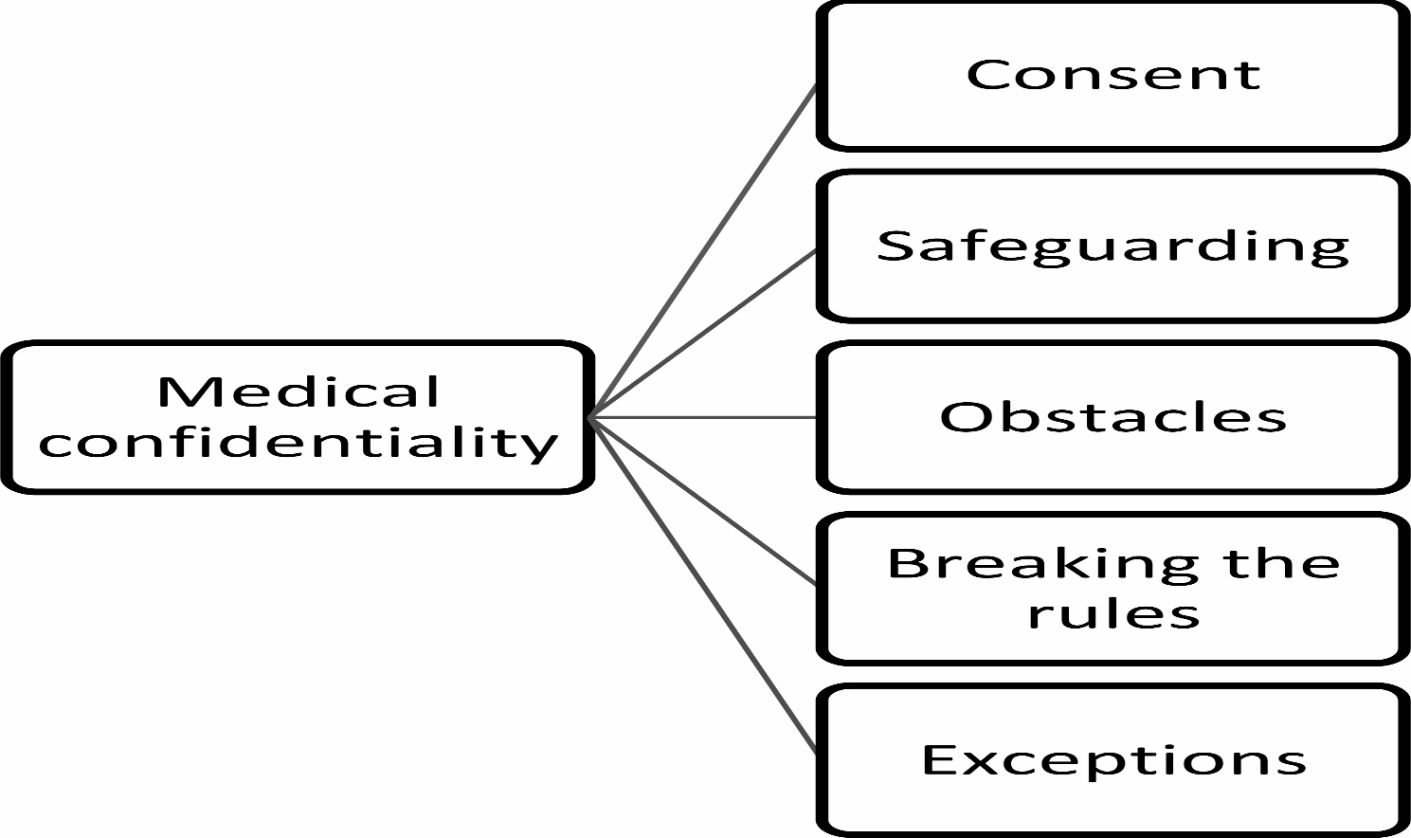
Themes of medical confidentiality

During a comprehensive study on responsiveness and dignity in Israel, physicians discussed how they and their teams handle medical information daily. Physicians raised concerns that can be defined along the following five themes: consent, safeguarding, obstacles, breaking the rules, and exceptions. Figure 1 presents the five themes of physicians’ concerns.

### Consent (to disclose/share information)

The analysis highlights that confidentiality is the act of protecting medical information from unauthorized access or disclosure [13, 17]. Healthcare providers have the legal and ethical responsibility to safeguard patients’ personal and medical information and prevent it from being disclosed to unauthorized individuals or entities without the patient’s consent:

To maintain medical confidentiality is to ensure that information and the knowledge does not reach anyone beyond those who are authorized to receive the medical information and, even then, only with the patient’s consent. (Interviewee #3).

The interviewees stressed that information should be protected from strangers and all unauthorized individuals:

When entering a room of four patients, we ask the whole family to leave the room because we cannot allow anyone to receive information about another patient. (Interviewee # 10).

Obtaining explicit consent from patients before disclosing information, even to family members, is crucial for building trust, promoting open communication, and protecting patient privacy:

If the son or daughter comes up to me to talk, then I tell them- I talked to mom, dad, aunt, or whoever it is. I talked to the patient and explained everything to him. So, you need to contact him, and he (i.e., the patient) will explain. … This way, I don’t feel that I am providing information behind (the patient’s) back (Interviewee # 11).

### Safeguarding medical data

Safeguarding medical information is critical in hospitals. However, structural limitations can challenge securing medical data and ensuring patient confidentiality. In the interviews, physicians shared innovative approaches to safeguarding patient privacy:

Even in conversations that occur in shared rooms, I try to ensure that the conversation is not in the presence of a person other than the patient or someone who cannot be taken out of the room. Like the neighbor. However, if there are family members of the neighbor, we ask them to wait outside. This, for me, is one of the basic requirements so that neither medical information is exposed, nor I harm the patient’s respect, dignity, and privacy. (Interviewee #5).

Safeguarding medical information can be achieved by raising awareness of the possibility of information leakage in public areas:

We make sure not to talk about a patient in the hallway. Make sure no ears are listening to us. (Interviewee # 10).

Physicians carefully choose their wording when writing official documents to avoid revealing unnecessary personal information:

We need to write many letters, for example, to insurance companies because the patient sometimes asks something from the insurance company, and they ask for a letter with a list of all the diseases. I prefer to use very general and confidential wording to prevent unwanted exposure. (Interviewee #3).

The strictness of patient data protection is also reflected in the concealment of patients’ names and identification details.

During the first accreditation (i.e., JCI accreditation: an American civil society organization that audits patient safety and quality of care), placards appeared in the corridors, warning doctors not to conduct corridor conversations. And not to mention patients at all. Even on the phone. Now, the list of patients is printed for you, and the ID numbers no longer appear there. No IDs; Only the patient’s first name or initials. (Interviewee #5).

The physicians who were interviewed for the study emphasized that all medical information nowadays is protected through the hospital system. This means patient records, test results, and other sensitive information are accessible only to authorized healthcare professionals:

We have been instructed in recent years that we are not allowed to transfer or make screenshots of our system or transfer information by WhatsApp for fear of hacking. All information is transferred only through our computer system. (Interviewee # 16).

Physicians describe high levels of data protection, including password-protected computers and shredded documents. The rise in the protection of patient’s medical information is reflected not only in the protection of written medical information but also in the provision of information via telephone conversations:

Nowadays doctors are more aware of whom to give information … Undoubtedly providing information over the phone is problematic. (Interviewee #6).

### Obstacles to protecting confidentiality

Keeping medical information confidential in a hospital setting is not an easy task but rather a complex issue [23, 28]. This is so because many individuals are exposed to both oral and written medical information despite the medical staff’s desire and commitment to protect it:

Many times, what happens or can happen in this situation, because of lack of time and so on, the other patient in the room or the family in the room will hear (confidential information). It happens. (Interviewee #8).

The public sphere seems to be the Achilles heel of keeping medical information confidential. Exposing sensitive medical information is a risk when patients are received in public areas or share rooms with others during visits [23, 24]:

I think the main obstacle in maintaining privacy is the doctors’ visit that takes place twice a day. This is how information is passed on to everyone. During the doctor’s visit, the neighbor is in the room. There is also a story of nurses’ shifts being transferred three times a day. And they do the same thing. In the end, it is very difficult to maintain privacy of information or medical confidentiality in a hospital setting. You can conduct the conversation privately, but in the end, there is no secrecy from those around you. (Interviewee #11).

Corridor conversations between physicians or with family members increase the chance of disclosing confidential information:

No doubt the hallway conversations do not help (i.e., privacy). This hurts the patient’s confidentiality … This is problematic. This is a sensitive issue. (Interviewee #6).

Unintentional exposure of patients’ medical information occurs due to a lack of training and knowledge of privacy practices:

I have personally seen some situations where families call, and you provide information over the phone. Not just me. Friends too… it is unintentional … and I am sure everyone makes that mistake, at least in the beginning. (Interviewee # 19).

### Breaking the rules (of confidentiality)

Technological advancements have significantly increased the ease of sharing information, including medical information (i.e., Electronic Health Records (EHR), Electronic Medical Records (EMR), Personal Health Records (PHRs), and Health Information Exchange (HIE) [31–39]). While these technologies enhance healthcare quality, safety, and efficiency by providing real-time, patient-centered records and facilitating provider coordination, unauthorized technological tools can pose security concerns. During the study, physicians repeatedly mentioned some unauthorized technology, such as WhatsApp groups, that are often used to share medical information. Although physicians are aware that this can potentially lead to the disclosure of sensitive data, they choose to ignore the rules:

At the subconscious level, we are aware that we must maintain the patient’s privacy (information). However, I do not think there is a difficult problem here other than technical matters. For example, we have a WhatsApp group, and sometimes medical information is discussed there. (Interviewee # 10).

Physicians often find themselves in a challenging position where they need to access and share data quickly and efficiently in order to provide the best possible care to their patients:

We do not protect anything at all. …. For example, I have a patient who has lupus and some other serious illnesses. So, I work with a nephrologist who works at another hospital. So, the other doctor and I are filming each other’s information and sending it on WhatsApp, like blood tests. This is to streamline the discussion and treatment for the patient. …. In this respect, this is probably problematic. (Interviewee # 10).

Another example of breaking the confidentiality rule is sharing information with family members without explicit consent. The decision to share information with family without ensuring consent can be linked to a certain mentality that values family above all else. It may also be linked to a lack of resources, such as a heavy workload or limited time, making it difficult to obtain proper consent before sharing information.

I think in this issue, we have too much openness, and if someone from the family comes, then because it is Israel, we do not go into the relationship with his brother. And the truth is that it is also impossible regarding the system. If you say, my dad or uncle, we share information, and we can certainly fail here, but we do not have the ability or resources to deal with it. (Interviewee #8).

In some cases, physicians may feel that they are acting in the family’s best interests by sharing information, even if it means bypassing the patient’s consent:

When we talk about patients who cannot make the decisions, we tend to share with family members. (Interviewee # 16).

Some interviewees even found it ridiculous to assume that medical information was not automatically shared with family members:

Obviously, we give information to families! Obviously, families are a very involved part of the treatment, although it is not exactly by law because that is the reality. (Interviewee # 20).

### Exceptions (to sharing information)

Physicians perceive sharing medical information among medical staff members as necessary to provide the patient with the best medical care possible. Without the transfer of information between doctors, promoting and providing quality medical care is difficult, if not impossible [13, 31, 32]. Staff collaboration expands knowledge, advances professionalism, and, therefore, advances the delivery of quality treatment to patients:

I have a patient with a very, very complex medical condition. .and very life-threatening condition. A young woman aged 42. She is an Arab woman from a village with a low socio-economic level; she does not speak Hebrew well and is truly from a weakened population. Her disease affects the heart, among other things. Therefore, I work in collaboration with a Heart Institute. The institute is involved in treatment from several disciplines of heart and a heart surgeon. We each bring a different angle. (Interviewee # 12).

## Discussion

The findings presented in the results section lend firm support all three hypotheses. Physicians present medical confidentiality as a legal and ethical obligation of healthcare providers to protect patients’ personal and medical information from being disclosed to unauthorized individuals or entities without patients’ consent [4]. This means that healthcare professionals are prohibited from disclosing information regarding a patient’s medical condition, treatment, or history. The emphasis is receiving the patient’s consent to disclose information [1–4]. Physicians highlight that patients have the right to control the ways that their personal information is shared and used, and healthcare providers must respect their wishes. Obtaining patients’ consent is viewed as respecting patients’ autonomy and promoting trust between patients and healthcare providers, both of which are essential for effective healthcare delivery [1, 2]. Physicians stressed the importance of safeguarding information and provided concrete examples of how they do so in practice. Hospitals can take several steps to secure patients’ medical information [23]. Strict access controls can limit medical record access to authorized personnel. Hospitals can use encryption and secure communication channels to protect medical information from unauthorized interception [31–39]. Training hospital staff in healthcare law and ethics is essential. Emphasizing ethical aspects of healthcare can increase the awareness of the medical teams not to disclose personal information in the presence of unauthorized persons or in public areas of the hospital, such as corridors or elevators [23, 24].

Notwithstanding the wide agreement regarding the importance of confidentiality and the strong desire to protect patients’ information in hospital settings, physicians described numerous challenges in protecting patient information. The public sphere, which includes public places such as hospital rooms and corridors, is often considered the Achilles heel of the public system when it comes to maintaining the confidentiality of medical information [23, 24]. This is a cause of concern for medical professionals who stress the importance of enhancing protection in those regions to ensure patient privacy (Hypotheses 2 and 3). Therefore, it is imperative that adequate measures are taken to safeguard the confidentiality of medical information in the public sphere [23].

The analysis on the subject brought to light two scenarios where physicians admitted breaching the rule of confidentiality by sharing information. In both cases, the physicians cited that they had to share the information to ensure that the patient’s well-being was not compromised and to maintain continuity of care. However, the first instance was presented as “breaking the rules”, suggesting that it was not the norm or that it was frowned upon. On the other hand, the second instance was presented as an “exception to the rule”, which suggests that sharing information under such circumstances was acceptable and justified. Hence, there are situations where it is necessary (and acceptable) to deviate from the confidentiality rule [27] (Hypothesis 1). For example, technological advancements have made sharing information easier [5, 31–39]. While this can certainly promote quality of care, it also raises concerns. For instance, using WhatsApp groups to transmit medical information can potentially lead to the disclosure of sensitive and personal data. Indeed, there is a thin and problematic boundary between information sharing for promoting medical treatment and unnecessary exposure.

Maintaining confidentiality in medical settings involves a series of trade-offs and challenges. Risks from providing secure and confidential elements can lead to an overemphasis of security, which may hinder, in turn, information sharing among healthcare providers and potentially negatively impact patient care. Additionally, complex security systems might increase the likelihood of user errors, leading to accidental breaches. Potential losses when achieving confidentiality can include crucial time lost, as implementing secure systems and protocols can be time-consuming. Moreover, staff training on confidentiality procedures takes time away from patient care, and accessing and sharing information securely may take longer than less secure methods. Therefore, maintaining medical confidentiality can be costly in terms of both finances and time. However, it is important to consider that these costs must be balanced against the risks of breaching confidentiality, which can lead to legal repercussions, harm to patients, and loss of patient trust. In conclusion, while upholding medical confidentiality is crucial, it does entail significant costs and potential trade-offs. Healthcare organizations need to find a balance between safeguarding patient privacy and ensuring efficient, effective care delivery.

## Health policy recommendations

In 2005, a few Israeli hospitals entered a voluntary process to obtain an international quality accreditation approval mark from the JCI^2^, an American civil society organization focusing on auditing patient safety and quality of care. In 2012, several years after the initiation of this process, the Director General of the Ministry of Health announced that all hospitals in Israel must obtain accreditation (Circular 38/2012) and conditioned the hospital’s license renewal and budget on receiving the accreditation mark. The accreditation process involves 1300 standards across 16 categories established by the JCI and a detailed section on medical confidentiality. Since then, most Israeli hospitals have obtained JCI accreditation and the medical staff are obligated to them.

One could assume that with so many measures in place, compliance with medical confidentiality would be high. Nevertheless, the discussion presented in this paper emphasizes that despite recognizing and knowing the theoretical importance of medical confidentiality, the rules are not always followed in practice. Hence, there is a wide gap between understanding and upholding the rules. Simply saying medical confidentiality is important is insufficient to promote it; hospitals need adequate structures to encourage compliance. Partial compliance with legal norms, such as medical confidentiality, can negatively impact an organization, such as legal liability, damage to reputation, and harm to the well-being of patients.

Therefore, in future healthcare policies, it is critical to ensure robust measures are in place to safeguard and uphold medical confidentiality. These can include specific measures to increase compliance, such as regularly monitoring compliance with confidentiality policies and enforcing consequences for any breaches to ensure accountability. For example, in order to uphold the monitoring and enforcement of confidentiality policies, hospitals can apply the following:

1. Conduct random audits to ensure compliance with confidentiality policies. Implementing random checks can help identify any breaches or potential areas of non-compliance.
2. Provide comprehensive training to all employees on confidentiality policies and procedures. This can help raise awareness and understanding of the importance of confidentiality and the consequences of breaches.
3. Establish clear and anonymous channels for employees to report any potential breaches or concerns regarding confidentiality. Creating anonymous voice mechanisms within the organization can promote employees’ and patients’ voices and help identify breaches or potential areas of non-compliance [40].
4. Clearly outline the consequences for breaching confidentiality policies and ensure that these consequences are consistently enforced. This can include disciplinary actions, termination of employment, or legal repercussions as appropriate.
5. Periodically review and update confidentiality policies to reflect changes in regulations, technologies, and practices. This ensures that the organization’s approach to confidentiality remains robust and relevant.

To address the complexity of physicians sharing sensitive patient information on various technological platforms, hospitals can apply several additional recommendations for future policies:

1. Implementation of secure messaging platforms such as encrypted messaging apps specifically designed for healthcare professionals.
2. Encouragement of the use of HIE or EHR systems [31–39] that allow authorized physicians from different institutions to access relevant patient data securely.
3. Enforcement of additional cybersecurity protocols, such as regular security audits, to detect and fix system vulnerabilities. All sensitive patient data should also be encrypted to prevent unauthorized access [41–43].

By implementing these measures, organizations can strengthen their monitoring and enforcement of confidentiality policies, ultimately fostering a culture of accountability and trust. Conversely, failing to implement medical confidentiality measures results in having guidelines that are merely theoretical and do not effectively translate into practice.

## Conclusion

Physicians face numerous challenges in protecting patient information despite recognizing the importance of confidentiality and the strong desire to protect information. Evaluating every circumstance individually is crucial and deciding wisely when and how to deviate from safeguarding information. Establishing precise regulations and policies can assist healthcare practitioners in ambiguous situations. Organizations can enhance their monitoring and enforcement of confidentiality policies by bolstering compliance.

## Appendix

**Table A: Physician’s characteristics**

**Table:** 

Number	Seniority*	Gender	Position**	Experience (in years)
1	Senior physician	Female	Management role	30
2	Senior physician	Male	Internal medicine	24
3	Senior physician	Male	Internal medicine	46
4	Academia and Senior	Male	Management role	36
5	Senior physician	Male	Internal medicine	30
6	Senior physician	Male	Internal medicine	40
7	Senior physician	Male	Management role	15
8	Senior physician	Male	Internal medicine	21
9	Senior physician	Male	Internal medicine	37
10	Senior physician	Male	Internal medicine	24
11	Senior physician	Female	Internal medicine	27
12	Senior physician	Male	Internal medicine	29
13	Senior physician	Female	Internal medicine	28
14	Senior physician	Female	Internal medicine	14
15	Other	Female	Internal medicine	8
16	Other	Female	Internal medicine	6
17	Senior physician	Male	Internal medicine	41
18	Others	Female	Internal medicine	6
19	Others	Female	Internal medicine	4
20	Academia and senior	Male	Management role	21

* Senior = senior physician or head of departments or senior management. Others = young physician and interns

** Internal medicine = active hospital physicians for all internal disciplines; Management = physicians in hospital management roles

## Appendix B: An ordered list of steps based on the thematic analysis process

1. Initial exploration:
  Conduct interviews.
  Take manual analytical notes throughout the research process.
2. Coding phase:
  Perform deep exploration of qualitative data.
  Develop surface meaning codes.
  Create interpretive codes.
  Capture participants’ perceptions of medical confidentiality.
3. Theme development:
  Organize coded data into potential sub-themes.
  Group sub-themes into broader themes.
4. Peer review and refinement:
  Discuss codes and themes with colleagues (as experts).
  Refine themes based on expert feedback.
  Consolidate into four main themes.
5. Verification:
  Review original interviews.
  Ensure codes and themes form a consistent and logical pattern.
6. Final documentation:
  Document final findings using: (a) Themes (b) Relevant analytical notes (c) Data excerpts.
  Ensure transferability and credibility of finding.

## Appendix C: Summary of key findings

**Table:** 

General Consensus	Medical confidentiality is a fundamental aspect of healthcare responsiveness and patient dignity	[1, 2, 10–13]
Doctor-Patient Relationship	Trust in the doctor-patient relationship is built on confidentiality	[3]
Sharing Information	Sharing medical information without consent breaches confidentiality	[3, 4]
Challenges in Modern Hospitals	Maintaining confidentiality is difficult due to multiple medical teams and electronic advancements Violations in public areas include disclosing patient data to unauthorized parties, discussing patient information publicly, and improperly disposing of records	[5–7, 22–24, 31–39]
Information Sharing	Information may be shared with other healthcare professionals involved in the patient’s care or with family members with a legitimate interest	[9]
Patient Requests for Privacy	Patients have the right to request that their medical information be kept private	[4]
Patient Perceptions of Confidentiality	Patients express concerns about information sharing and protection	[1, 8]
Physician Perspectives on Confidentiality	Physicians have varying views on when to breach confidentiality	[27, 28]
Balancing Confidentiality and Public Safety	There are instances where breaching confidentiality may be necessary to protect public safety or patient well-being	[7]
Benefits of Sharing Information	Sharing more comprehensive information can lead to better patient care	[10–12]
Responsiveness	Comprehensive information can lead to a more responsive health system	[10–16]
Local legislation	Local Israeli laws allow for breaches of confidentiality to protect public safety or patient well-being	[25, 26]
Awareness/legal consciousness	Patients and healthcare workers are not always aware of medico-legal issues and rights	[1, 20]

## Data Availability

The data supporting this study’s findings are available on request from the corresponding author.

## Footnotes

1 For an ordered list of research steps, see Appendix B.

2 The Joint Commission International (JCI) is a global organization that accredits and certifies healthcare organizations worldwide. The JCI sets international standards for quality and patient safety in healthcare delivery.
